# Increased Avidity of the *Sambucus nigra* Lectin-Reactive Antibodies to the Thomsen-Friedenreich Antigen as a Potential Biomarker for Gastric Cancer

**DOI:** 10.1155/2015/761908

**Published:** 2015-11-18

**Authors:** Oleg Kurtenkov, Kersti Klaamas

**Affiliations:** Department of Oncology and Immunology, National Institute for Health Development, Hiiu 42, 11619 Tallinn, Estonia

## Abstract

*Aim*. To determine whether the naturally occurring Thomsen-Friedenreich (TF) antigen-specific antibodies differ in avidity between cancer patients and controls to find a novel biomarker for stomach cancer.* Methods*. Serum samples were taken from patients with cancer and controls. The level of TF-specific antibodies and their sialylation were determined using ELISA with synthetic TF-polyacrylamide conjugate as antigen and sialic acid-specific* Sambucus nigra* agglutinin (SNA). The avidity was determined using ammonium thiocyanate as a chaotrope.* Results*. A significantly higher SNA lectin binding to anti-TF antibodies was found in cancer patients irrespective of disease stage. The avidity of only IgM TF-specific antibodies was significantly higher in cancer patients compared to controls. The SNA-positive anti-TF antibodies of cancer patients showed a significantly higher avidity, *P* < 0.001. The sensitivity and specificity of this increase for gastric cancer were 73.53% and 73.08%, respectively, with a 73.2% diagnostic accuracy. The higher avidity of SNA-reactive anti-TF antibodies was associated with a benefit in survival of stage 3 cancer patients.* Conclusion*. The SNA-reactive TF-specific antibodies display a significantly higher avidity in gastric cancer patients compared to controls, which can be used as a potential serologic biomarker for gastric cancer. It appears that IgM is the main target responsible for the above changes.

## 1. Introduction

Over the past two decades protein posttranslational modifications have attracted ever-increasing attention in medical research. The altered immature O-glycophenotype often observed in cancer cells leads to the expression of modified glycopeptide epitopes and tumor-associated glycans (TAGs) that may be autoimmunogenic and recognized by autoantibodies [1–9]. In cancer patients, an abnormal glycosylation pattern has also been observed for many circulating glycoconjugates, including immunoglobulins [10–15].

The O-linked tumor-associated glycans such as the Thomsen-Friedenreich (TF) antigen (Gal*β*1-3GalNAc*α*/*β*-O-Ser/Thr, TF, CD176) and Tn antigen (GalNAc*α*1-O-Ser/Thr, CD175) are expressed in the majority of human carcinomas [1, 16–18], including cancer-initiating cells [19]. TAGs are considered as a promising target for cancer immunotherapy [20–23]. The overexpression of these commonly hidden glycotopes and the reduced level of naturally occurring anti-TF or anti-Tn antibodies are associated with tumor progression and aggressiveness and a patients survival rate [16, 24–29]. The TF antigen seems to play a crucial role in the adhesion of cancer cells to the endothelium through the interaction with galectin-3, thereby promoting metastases [30, 31].

The presence of autoantibodies (AAbs) against antigens expressed by tumors, including TAGs, is a well-established fact [4]. However, a majority of AAbs to tumor-assocoated antigens (especially anti-peptide Abs) is revealed only in a minority of cancer patients, thus limiting the clinical potential of the approach. An appreciable amount of TF- and Tn-specific IgM and IgG antibodies is present in normal human serum, being decreased in patients with cancer though there are large interindividual variations [17, 25–27]. Moreover, the anti-TF and -Tn IgG level is rather stable over time at an individual level in both patients and controls [25, 32]. However, the antitumor potential of tumor-specific Abs remains to be further elucidated because the latter may actually have various effects [8, 21, 33, 34], suggesting that these antibodies are heterogeneous functionally and structurally. Of note is that up to now there are very limited data available on the glycosylation of naturally occurring human TAG-specific Abs [15, 35–37] and, to our knowledge, no data about the avidity profile of these antibodies in cancer or other pathologies have been reported either. Gastric cancer is the second leading cause of cancer deaths worldwide. Yet there are no reliable serum biomarkers for gastric cancer diagnostics and prognostics.

We showed recently that patients with gastric cancer demonstrated an increased sialylation of TF-specific Abs irrespective of disease stage, tumor morphology, and gender [36]. Moreover, we found that similar changes in anti-TF Ab sialylation were also observed in patients with breast cancer (unpublished), suggesting that it may be a common cancer-related phenomenon. In the present study we show, for the first time, that gastric cancer is associated with a significantly higher avidity (*P* < 0.001) of SNA-positive TF-specific antibodies that may be used as a serologic biomarker for gastric cancer.

## 2. Material and Methods

### 2.1. Subjects and Samples

**Figure 1:**
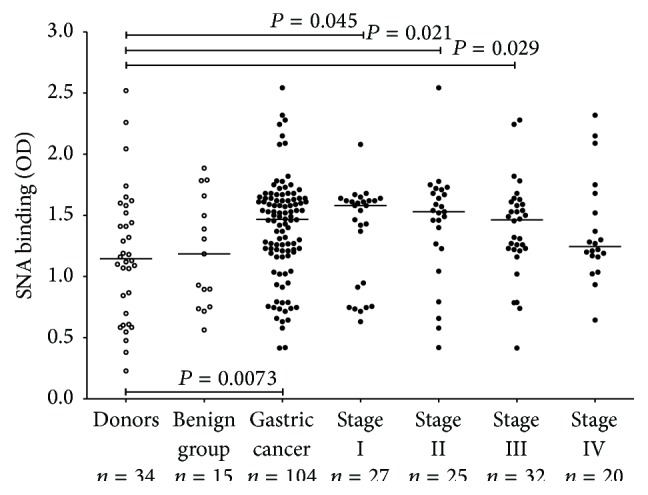
The binding of* Sambucus nigra* agglutinin (SNA) to serum TF-specific antibodies (all isotypes) in controls and gastric cancer patients by stage of disease. Each dot represents one individual and group median is indicated by horizontal lines. *P* values were calculated by the Mann-Whitney *U* test and are shown for significant differences.

Serum samples were obtained from healthy blood donors (*n* = 34), patients with nonmalignant gastric diseses (*n* = 15), and patients with histologically verified gastric carcinoma (*n* = 104) (Table 1). Tumor staging and morphology were based on the histopathological (pTNM) classification of malignant tumors. The distribution of cancer patients by stage is presented in Figure 1. The investigation was carried out in accordance with the ICH GCP Standards and approved by the Tallinn Medical Research Ethics Committee. A written informed consent was obtained from each subject. The serum samples were stored in aliquots at –20°C until used.

### 2.2. The Anti-TF Antibody Assay

The anti-TF IgG, IgM, and a pool of IgG+IgM+IgA antibody levels were determined by enzyme-linked immunosorbent assay (ELISA) as described elsewere [36]. The plates (Maxisorp, NUNC, Denmark) were coated with synthetic TF-polyacrylamide conjugate (10 mol% of carbohydrate; Lectinity, Russia) in carbonate buffer, pH 9.6, 5 *μ*g per well. After overnight incubation at +4°C, triple washing and blocking with Superblock solution (Pierce, USA) for 30 min at 25°C, the serum samples (diluted 1 : 25 in PBS-0.05% Tween) were applied for 1.5 hr at 25°C. After subsequent washing with PBS-Tw, the bound anti-TF antibodies were detected using alkaline phosphatase conjugated goat anti-human IgG, IgM (Sigma, USA), IgA (Dako, Denmark), or rabbit anti-IgG+IgM+IgA (Dako) and developed with p-nitrophenylphosphate (Sigma, USA). The absorbance values were read at 405 nm (Tecan Reader, Austria) and each sample was analysed in duplicate.

### 2.3. The Reactivity of Anti-TF Antibodies to* Sambucus nigra* Agglutinin (SNA)

The SNA lectin-reactivity of TF-glycotope specific antibodies was measured in a similar way.

The plates (Maxisorp, NUNC, Denmark) were coated with synthetic TF polyacrylamide conjugate (10 mol% of carbohydrate; Lectinity, Russia) in carbonate buffer, pH 9.6, 5 *μ*g per well. After overnight incubation at +4°C, triple washing and blocking with Superblock solution (Pierce, USA) for 30 min at 25°C, the serum samples (diluted 1 : 25 in PBS-0.05% Tween) were applied for 1.5 hr at 25°C. After subsequent washing with PBS-Tw, the biotinylated SNA (Vector Laboratories Inc., USA) in 10 mmol/L Hepes, 0.15 mol/L NaCl, 0.1 mmol/L CaCl_2_, and pH 7.5 was applied at a concentration of 5 *μ*g/mL for 1.5 hr at 25°C. The bound lectin was detected with a streptavidin-alkaline phosphatase conjugate (Dako, Denmark) and p-nitrophenylphosphate (Sigma, USA). The optical density value (OD) of control wells (no sample) was subtracted from the Ab coated wells. Each sample was analysed in duplicate.

### 2.4. The Avidity of TF-Specific Antibodies

The avidity of anti-TF IgG, IgM, or a pool of IgG+IgM+IgA antibodies was determined by ELISA. The plates were coated with synthetic TF-polyacrylamide conjugate in carbonate buffer, pH 9.6, 5 *μ*g per well. After overnight incubation at +4°C, washing and blocking with Superblock solution as above, the serum (diluted 1 : 25 in PBS-0.05% Tween) was applied for 1.5 hr at 25°C. After subsequent washing ammonium thiocyanate (NH_4_SCN) as a dissociating agent was added at a concentration of 1.25 mol/L for 15 min at +25°C. The bound antibodies were detected with alkaline phosphatase conjugated goat anti-human IgG, IgM or anti-(IgG+IgM+IgA) Igs, and p-nitrophenylphosphate. The absorbance values were read at 405 nm.

A relative avidity index (AI) was calculated for each sample and was expressed as the percentage of reactivity remaining in the thiocyanate-treated wells in relation to untreated wells (PBS-Tw instead of the chaotrope).

### 2.5. The Avidity of* Sambucus nigra* Agglutinin- (SNA-) Reactive Anti-TF Antibodies

The avidity of SNA-reactive anti-TF antibodies (a pool of all isotypes) was determined by ELISA in a similar way. The plates (Maxisorp, NUNC, Denmark) were coated with synthetic TF polyacrylamide conjugate as above. After overnight incubation at +4°C, triple washing and blocking with Superblock solution for 30 min at 25°C, the serum samples (diluted 1 : 25 in PBS-0.05% Tween) were applied for 1.5 hr at 25°C. After subsequent washing ammonium thiocyanate (NH_4_SCN) as a dissociating agent was added at a concentration of 1.25 mol/L for 15 min at +25°C. To detect the lectin reactive antibodies, the biotinylated SNA (Vector Laboratories Inc., USA) in 10 mmol/L Hepes, 0.15 mol/L NaCl, 0.1 mmol/L CaCl_2_, and at pH 7.5 was applied at a concentration of 5 *μ*g/mL for 1.5 hr at 25°C. The bound lectin was detected with a streptavidin-alkaline phosphatase conjugate and p-nitrophenylphosphate. The proportion of TF-specific antibody SNA reactivity remaining after treatment with chaotrope was defined as the avidity index of SNA-reactive anti-TF antibodies.

### 2.6. Statistical Analysis

The results were analysed using the nonparametric Mann-Whitney *U* test due to the abnormal distribution of values. The difference between the groups was considered to be significant when *P* ≤ 0.05. The sensitivity and specificity of the differences between cancer patients and controls were evaluated by the receiver operator characteristic (ROC) curve analysis. Overall survival was analyzed by the Kaplan-Meier method. All calculations and comparisons were performed using GraphPad Prism 5 and SPSS 15.0 software.

## 3. Results

The levels of anti-TF IgG in cancer patients and both controls were very similar and were decreased only in patients with advanced cancer: mean O.D. = 0.50 ± 0.05 (SE) and 0.36 ± 0.05 (stage 4), *P* = 0.018 compared to healthy donors.

In a parallel testing of several anti-TF Ab isotypes (IgG, IgM, IgA, and a pool of all isotypes) only IgM showed a clear trend to a lower level in cancer (*n* = 36) compared to healthy donors (0.22 ± 0.07 (SD) and 0.31 ± 0.2, resp., *P* = 0.08) and a significant decrease compared to the benign group (0.37 ± 0.12, *n* = 15, *P* < 0.001).

The SNA lectin binding to serum TF-specific antibodies (all isotypes) was significantly higher in cancer patients compared to controls (*P* = 0.0073) (Figure 1). The stage distribution had no impact on this increase, except in stage 4 patients that showed no significant changes in SNA binding compared to controls.

**Figure 2:**
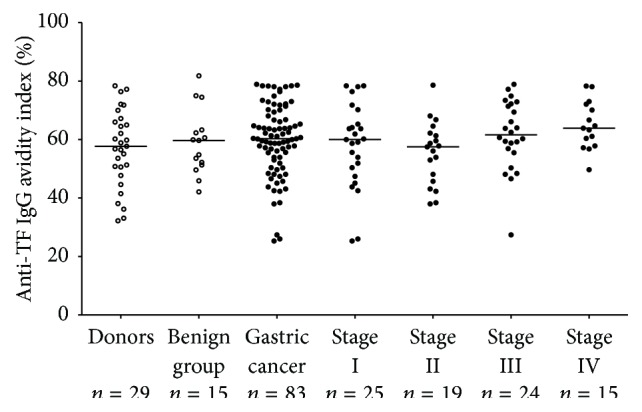
The avidity of anti-TF IgG in controls and patients with gastric cancer by stage.

The avidity of anti-TF IgG reveals no significant differences between cancer patients and controls (59.7 ± 12.2 (SD) and 57.1 ± 13.1, resp.) with no significant changes by stage of the disease (Figure 2), being in the range of 56–64%.

A group of cancer patients, blood donors, and patients from the benign group were tested in parallel for the avidity of anti-TF IgG, IgM, and a pool of IgG+IgM+IgA anti-TF Abs (Table 2). The avidity of only IgM TF-specific Abs was significantly higher in cancer compared to both controls (*P* = 0.002 and *P* < 0.0001 for donors and the benign group, resp.), suggesting that the anti-TF IgM is the main target for changes in the TF-specific Ab avidity found in cancer patients. Interestingly, patients with nonmalignant gastric diseases showed an even lower level of Ab avidity than healthy blood donors.

**Figure 3:**
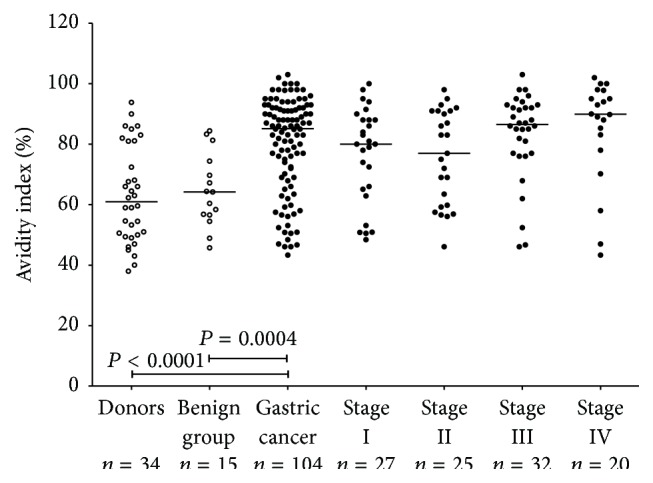
The avidity of SNA-positive anti-TF antibodies in controls and patients with gastric cancer by stage. *P* values were calculated by the Mann-Whitney *U* test and are shown for significant differences.

**Figure 4:**
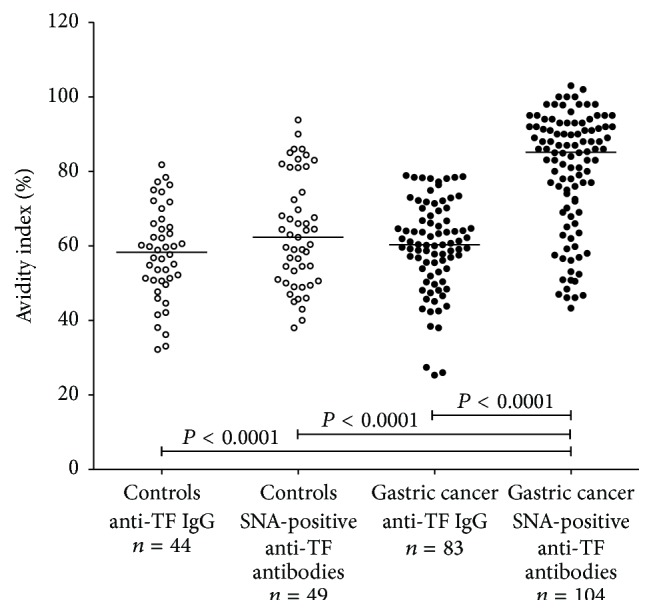
Comparison of the avidity of anti-TF IgG and SNA-positive TF-specific serum antibodies in cancer patients and controls. *P* values are shown for significant differences.

The avidity of SNA-positive anti-TF antibody (all isotypes) was significantly higher in patients with cancer compared to both control groups (*P* < 0.0001 and *P* < 0.0004 for donors and the benign group, resp.) with slightly higher avidity index values in advanced cancer (Figure 3). It is notable that in controls the avidity of anti-TF IgG was very similar to that of anti-TF IgG in cancer patients, whereas the avidity of SNA-positive TF-specific antibodies in cancer patients was significantly higher compared with that of the other groups of patients and controls (Figure 4). The higher avidity of SNA-positive anti-TF antibodies in cancer patients was not dependent on gender and/or age (data not shown).

**Figure 5:**
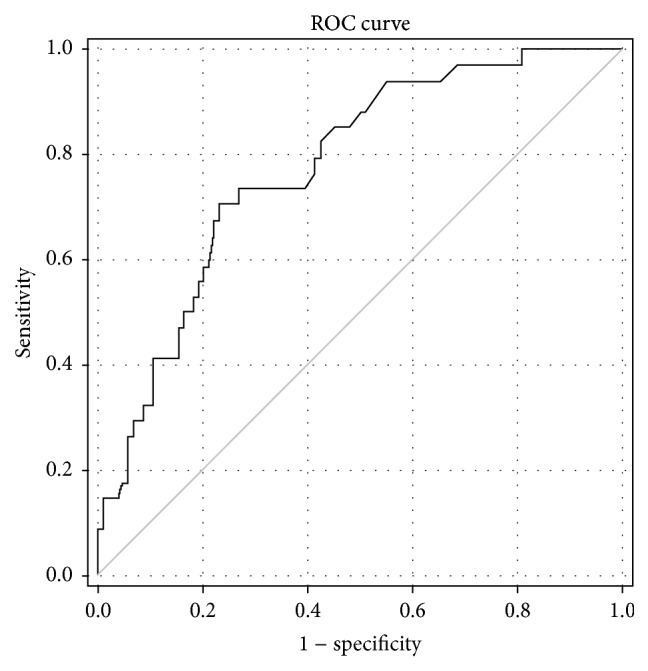
The sensitivity and specificity of serum anti-TF antibody avidity changes for gastric cancer by receiver operator characteristic (ROC) curve analysis.

Using the SNA-positive TF-specific antibody avidity value equal to 72.45% as a cut-off limit, which allows the best discrimination between cancer patients and controls (calculated by ROC curve analysis), the sensitivity and specificity for gastric cancer were 73.53% and 73.08%, respectively, with a 73.2% accuracy of diagnostics (ROC statistics: the area under a curve 0.776, *P* < 0.0001) (Figure 5). The sensitivity of the test was 70.37%, 60%, 84.37%, and 80% for stages 1, 2, 3, and 4, respectively. At a specificity of 90% the sensitivity was 47.9%.

**Figure 6:**
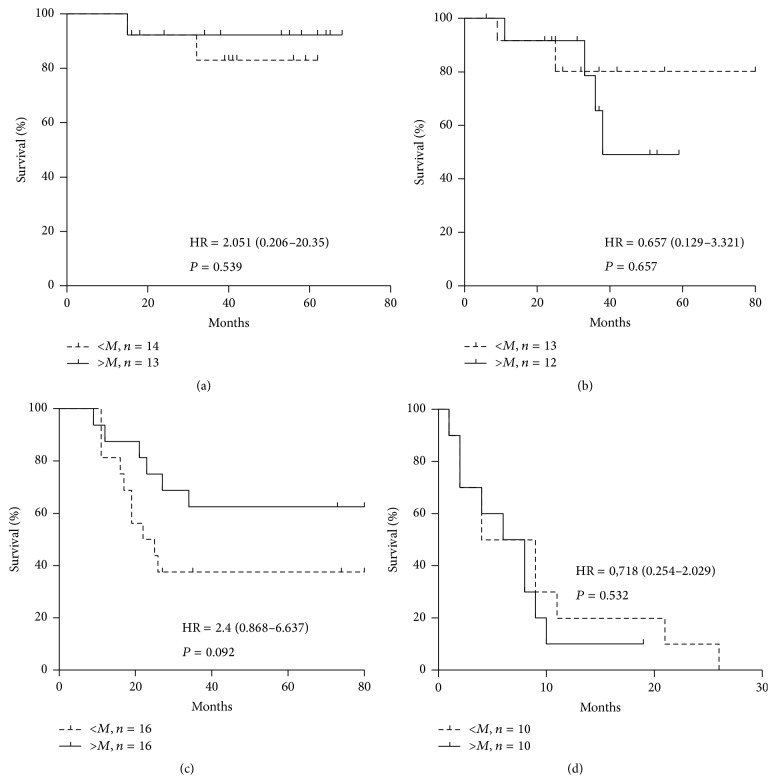
The probability of survival of gastric cancer patients in relation to the avidity of SNA-positive anti-TF antibodies. Patients with avidity index values lower than, equal to (a dashed line), or higher than median (a solid line) are compared using the Kaplan-Meier method. The hazard ratio (HR) with a 95% confidence interval and *P* values are shown. Patients: (a) stage 1; (b) stage 2; (c) stage 3; (d) stage 4.

For the whole group of cancer patients (all stages), no significant association of the avidity of anti-TF IgG antibodies with survival was found (HR = 0.72 (95% CI 0.38–1.37), *P* = 0.32). However, the higher avidity of SNA-reactive serum anti-TF antibodies was associated with a benefit in survival of stage 3 cancer patients (HR = 2.4 (0.86–6.36), *P* = 0.09) (Figure 6).

## 4. Discussion

An aberrant glycosylation of glycoconjugates, including immunoglobulins, is a common phenomenon in cancer [3, 5, 32, 38]. It has been shown that various Ab glycoforms display different effector functions and determine the activity of antibodies against tumors [5, 39–41]. Previous studies have demonstrated that naturally occurring antibodies to tumor associated glycans are involved in natural tumor immunity, being associated with tumor progression and cancer patients survival [18, 26, 42, 43]. Natural anti-TF antibodies (Abs) of different isotypes (IgG, IgM, and IgA) are present in each individual thus making these antibodies a convenient target for analysis, in contrast to tumor-derived products that may be detected in a minority of patients due to their extreme dilution in the circulation and rapid degradation or clearance. Although the levels of TF-specific antibodies demonstrate some decrease in cancer patients [7, 17, 26], these changes did not show sufficient sensitivity and specificity for gastric cancer [36].

We analysed the TF-specific Abs present in the whole untreated serum, using TF-PAA as a catcher and the* Sambucus nigra* agglutinin (SNA) in the lectin-ELISA assay, thus excluding possible structural or conformational modifications of Igs during their purification or the presence of so called “hidden” Abs that may remain undetectable due to being in complexes with some TF-positive ligands such as aberrantly glycosylated MUC1, for instance. We showed recently that the increase of SNA binding to TF-specific Abs in cancer patients was not dependent on the stage of disease, histological type of tumor growth (diffuse, intestinal), or gender [36]. In contrast, the SNA reactivity of anti-TF IgG in purified IgG samples was even decreased in patients with gastric cancer [15]. The higher level of the fully sialylated IgG glycoform, as defined by LC-ESI-MS, may predict a better survival of patients with gastric cancer [14].

In the present study we show that the avidity of serum anti-TF IgG is not increased in cancer, in contrast to that of IgM (Figure 2, Table 2), indicating that IgG is not involved in cancer-related changes of TF-specific antibody avidity. Preliminary data show that the IgG depletion does not influence significantly the SNA binding to all TF-specific Abs and the avidity index of SNA-reactive Abs in both donors and cancer patients (unpublished). It appears that the anti-TF IgM remain to be the main target responsible for that [36].

An increase in avidity of SNA-reactive anti-TF antibodies showed a rather good diagnostic accuracy (73.2%). Note that already stage 1 patients showed a relatively high diagnostic sensitivity (70.37%). At present, we can give no explanation for why the SNA-positive anti-TF Abs have a relatively high avidity only in cancer patients and not in donors. The increased SNA reactivity of anti-TF Abs in cancer indicates that they are more sialylated than those in donors. It has been shown that the SNA mostly bound the IgG Fc glycan at Asn297, which has two sialic acids if both glycan branches terminated with sialic acid [44]. The pentameric IgM is more glycosylated and has five N-glycosylation sites (four of which on Fab) on each of its heavy chains [5]. There are still no data about the sialylation diversity of pentameric IgM Fc and Fab in health and disease, and its influence on Ab avidity. It is possible that different proportions of IgM and/or IgA fully sialylated anti-TF Abs may be present in patients and controls. The IgM polyvalency and possible variations in the glycosylation of different monomers may also be infuencing factors. Another reason might be the increased activity of sialyltransferases that orchestrate the diversity of glycan structures and are frequently overregulated in cancer cells [45–47] and tumor microenvironment where TF-specific antibodies may interact with TF-positive tumor cells and undergo further sialylation* in situ*. But the question remains unanswered yet.

In any case, the higher avidity of serum SNA-reactive TF-specific Abs we observed in patients with gastric cancer needs further investigation from several points of view: (i) specifying the Ig isotype responsible for these changes, especially the site-specific glycosylation patterns; (ii) studying how this alteration in Ab sialylation influences the functional (antitumor) Ab activity; (iii) exploiting a combination of Ab avidity testing with other Ab-based parameters such as Ab levels, the diversity of other glycoforms, the presence of hidden Abs, and putative hidden antigens (ligands) in the circulation; (iv) further stratifying the patients on the basis of additional parameters, as has been done for SNA binding and anti-TF IgM level patterns in patients with gastric cancer [36].

Given that the expression of TF antigen on tumor cells promotes metastasis by interaction with galectin-3 on the endothelial cells [30], it is logical to assume that the circulating TF-specific antibody may modulate this interaction* via* binding with TF antigen-positive circulating tumor cells. It is not known yet whether the aberrantly glycosylated (sialylated) anti-TF antibodies interact differently with tumor cells* in situ* or in the circulation. Since the sialylated (anti-inflammatory) Abs display immunosuppressive or tolerogenic effects [37, 48], they may eliminate undesirable inflammatory reactions in tumor tissue that may promote tumor growth [8, 49]. Alternatively, a benefit in survival we found in stage 3 cancer patients with a higher avidity of TF-specific Abs might be the result of a more efficient blockade of TF antigen on circulating tumor cells, thus protecting against metastases through the inhibition of the TF/galectin-3 pathway. The other functional activities of aberrantly sialylated TF-specific antibodies remain to be determined.

In conclusion, we provide evidence that the aberrant sialylation of TF-specific antibodies is associated with gastric cancer-specific changes in Ab avidity, which may be used as a potential serological biomarker for gastric cancer detection and prognosis. It is to be noted that a rather high diagnostic sensitivity (70.37%) was observed already in stage 1 patients. Our data suggest that the evaluation of not just the level of antibodies to tumor-associated antigens but rather their structural and functional diversity might improve the clinical potential of antibody signatures in cancer diagnostics and prognostics. Since the glycosylation of Abs specific to various antigens may considerably differ from that of total Ab isotypes in the circulation, the focus of further studies should be shifted to the glycoprofiling of Abs specific to antigens directly involved in the pathogenesis of the disease under study. Such a noninvasive approach which is not yet widely used in practical medicine may be a good prerequisite for the improvement of the clinical utility of antibody-based biomarkers.

## Figures and Tables

**Table 1:** Characteristics of the subjects tested.

Group	*n*	Males	Females	Median age (range)
Donors	34	9	25	63 (24–73)
Benign group^x^	15	9	6	62 (27–72)
Gastric cancer	104	59	45	66 (28–80)

^x^Nonmalignant chronic gastric diseases: peptic ulcer disease (*n* = 9); chronic gastritis (*n* = 6).

**Table 2:** The avidity of anti-TF IgG, IgM, and a pool of all anti-TF Ab isotypes in gastric cancer patients and controls. The mean ± SD and *P* values are presented.

Groups	*n*	IgG	IgM	IgG/M/A
Donors	16	56.7 ± 14.8	44.9 ± 14.0	55.1 ± 7.1
Benign group	15	59.1 ± 11.2	39.9 ± 6.0	47.7 ± 6.7
Gastric cancer	36	59.9 ± 10.3	59.2 ± 12.2	54.4 ± 5.9

*P* values				
Donors versus benign group		0.61	0.22	0.01
Donors versus gastric cancer		0.43	0.002	0.75
Benign group versus gastric cancer		0.8	<0.0001	0.003
